# Canonical Notch signalling is inactive in urothelial carcinoma

**DOI:** 10.1186/1471-2407-14-628

**Published:** 2014-08-29

**Authors:** Annemarie Greife, Silvia Jankowiak, Jochen Steinbring, Parvaneh Nikpour, Günter Niegisch, Michèle J Hoffmann, Wolfgang A Schulz

**Affiliations:** Department of Urology, Medical Faculty, Heinrich Heine University Düsseldorf, Düsseldorf, Germany; Institute of Pathology, Medical Faculty, Heinrich Heine University Düsseldorf, Düsseldorf, Germany; Department of Genetics and Molecular Biology, Faculty of Medicine, Isfahan University of Medical Sciences, Isfahan, Iran

**Keywords:** Urothelial carcinoma, Notch pathway, γ-secretase inhibitors, Bladder cancer, NOTCH1

## Abstract

**Background:**

Notch signalling regulates cell fate in most tissues, promoting precursor cell proliferation in some, but differentiation in others. Accordingly, downregulation or overactivity variously contributes to cancer development. So far, little is known about Notch pathway activity and function in the normal urothelium and in urothelial carcinoma (UC). We have therefore investigated expression of Notch pathway components in UC tissues and cell lines and studied the function of one receptor, NOTCH1, in detail.

**Methods:**

Expression of canonical Notch pathway components were studied in UC and normal bladder tissues by immunohistochemistry and quantitative RT-PCR and in UC cell lines and normal cultured urothelial cells by qRT-PCR, immunocytochemistry and Western blotting. Pathway activity was measured by reporter gene assays. Its influence on cell proliferation was investigated by γ-secretase inhibition. Effects of NOTCH1 restoration were followed by measuring cell cycle distribution, proliferation, clonogenicity and nuclear morphology.

**Results:**

NOTCH1 and its ligand, DLL1, were expressed at plasma membranes and in the cytoplasm of cells in the upper normal urothelium layer, but became downregulated in UC tissues, especially in high-stage tumours. In addition, the proteins were often delocalized intracellularly. According differences were observed in UC cell lines compared to normal urothelial cells. Canonical Notch pathway activity in reporter assays was repressed in UC cell lines compared to normal cells and a mammary carcinoma cell line, but was induced by transfected NOTCH1. Inhibitors of Notch signalling acting at the γ-secretase step did not affect UC cell proliferation at concentrations efficacious against a cell line with known Notch activity. Surprisingly, overexpression of NOTCH1 into UC cell lines did not significantly affect short-term cell proliferation, but induced nuclear abnormalities and diminished clonogenicity.

**Conclusion:**

Our data indicate that canonical Notch signalling is suppressed in urothelial carcinoma mainly through downregulation of NOTCH1. These findings can be explained by proposing that canonical Notch signalling may promote differentiation in the urothelium, like in many squamous epithelia, and its suppression may therefore be advantageous for tumour progression. As an important corollary, inhibition of canonical Notch signalling is unlikely to be efficacious and might be counter-productive in the treatment of urothelial carcinoma.

**Electronic supplementary material:**

The online version of this article (doi:10.1186/1471-2407-14-628) contains supplementary material, which is available to authorized users.

## Background

Bladder cancer is the fifth most common malignancy in most Western industrialized countries with urothelial carcinoma (UC) representing the major histological subtype. UC can be further classified into papillary and invasive carcinomas [1]. While most cases of papillary low grade urothelial tumours have a good prognosis, high grade papillary tumours tend to progress towards invasive cancers which are often lethal. A large fraction of invasive urothelial carcinomas develop by a different route via high-grade dysplastic carcinoma in situ [2]. Depending on tumour stage, urothelial carcinoma treatment is treated either by transurethral resection or radical cystectomy. In addition neo-adjuvant or palliative chemotherapy is used in locally advanced or metastatic urothelial carcinomas, respectively [3, 4]. Local immunotherapy or chemotherapy is used for preventing recurrences of superficial cancers. Until now, clinical studies using molecular targeted drugs have yielded disappointing results [5–7]. This failure is generally considered to reflect our insufficient knowledge of urothelial tumour biology.

The Notch signalling pathway is a promising target for cancer therapy [8, 9]. In many tissues, Notch signalling contributes to the maintenance of stem cells or precursor cells [10] and pathway hyperactivation caused by point mutations [11] or translocations involving *NOTCH* genes [12, 13] is oncogenic by blocking cell differentiation, protecting against apoptosis and stimulating proliferation. Conversely, in certain epithelia, Notch signalling promotes differentiation. Accordingly, pathway inactivation, frequently caused by NOTCH1 mutations, is observed in squamous carcinomas of several organs [14–16]. Therefore, depending on tissue and tumour type, drugs targeting Notch signalling may be therapeutic, useless or tumour-promoting [17].

Notch signalling constitutes a short-range communication system between adjacent cells. Canonical Notch signalling is activated by ligand-receptor interactions releasing the receptor intracellular domain (NICD) by two proteolytic steps, including an essential cleavage by γ-secretase. The NICD translocates into the nucleus where it binds to C Promoter Binding Factor-1 (CBF-1), recruits co-activators (MAML1 and KDM5A) and typically activates the transcription of *Hairy and Enhancer of Split (HES)* and *Hairy and Enhancer of Split related (HEY)* target genes. Through such effectors Notch determines cell fate [18].

To date, little information exists on the Notch signalling pathway in normal urothelium or urothelial cancers. Decreased expression of several Notch receptors and ligands in immunohistochemistry has been reported especially in papillary UC [19]. We have investigated the expression of Notch pathway components in urothelial carcinoma tissues and cell lines and the function of NOTCH1 in UC cell lines.

## Methods

### Tissue samples

The benign and tumour bladder samples used for gene expression studies were a subset of those described previously [20] comprising 11 benign bladder tissues and 30 bladder cancer tissues from 25 male and 5 female patients with ages ranging from 54 to 84 years. Tumour stages and grading according to the UICC classification were as follows: pT3 G3 in 11 cases, pT4 G3 in 6 cases, pT2 G2 in 6 cases, pT2 G3 in 3 cases and one case each of pT3 G2, pT1 G2, pTa G3 and pTa G2 tumours. The set used for immunohistochemistry comprised 4 normal tissues, 27 UC tissues with 6 pTa low grade, 5 pTa high grade, 5 pT1, 3 pT2, 3 pT3, 4 pT4, 1 CIS. The study was approved by the ethics committee of the medical faculty of the Heinrich Heine University and informed consent was obtained from all patients.

### Cell lines and primary cultures

All UC cell lines (5637, 639v, 647v, BFTC905, HT1376, J82, RT4, RT112, SD, SW1710, UM-UC3, VM-Cub1, T24) and HEK293 cells were cultured in DMEM GlutaMax (Gibco, Darmstadt, Germany), supplemented with 10% fetal calf serum [21]. They were obtained from the DSMZ (Braunschweig, Germany), except for UM-UC3, kindly provided by Dr. Grossman, Houston. The well-differentiated UC cell line BC61 was cultured and characterized as previously described [22, 23]. Primary urothelial cells (UP) were prepared and maintained as described (20). T47D cells were maintained in RPMI1640 supplemented with 15% foetal calf serum and 10 μg/ml insulin.

### Plasmids

Human N1ICD-pIRES2 was a kind gift from Prof. Giebel, Essen; human full-length wildtype NOTCH1 in a pcDNA3-HA/FLAG vector was kindly provided by Prof. Di Fiore, IFOM, Italy [24].

### Gamma secretase inhibitor treatment

Cells were treated with 0.25 - 20 μM γ-secretase inhibitors DAPT (#2634, TOCRIS/R&D Systems), L-685,458 (#2627, TOCRIS/R&D Systems) or Compound E (#56597, Calbiochem/Millipore) or, as a control, compound W (#2654, TOCRIS/R&D Systems) for 48 h when viability was analysed by MTT assay.

### Transfection and reporter gene assay

For reporter gene assays, cells seeded in 6-well plates were transiently transfected with XtremeGene9 (Roche) with 1 μg of either pJH26A (Notch responsive reporter) or pJH28A (Notch non-responsive reporter), kindly provided by Prof. D. Hayward [25], or in other experiments either pHES1 (+CBF1)-luc (addgene 41723; similar to pJH26A) or pHES1 lacking the CBF1 site (addgene 43805, similar to pJH28A). p850-luc and pGL3 (Promega) were used as positive or negative controls, respectively. NOTCH1 expression plasmids were added as indicated in the individual experiments, using the respective plasmid vectors for adjusting total amounts transfected. All cells were co-transfected with 10 ng Renilla luciferase plasmid for normalization. Cells were harvested 48 h after transfection and assayed for luciferase activities by the Dual-Luciferase reporter assay (Promega). Notch activity was determined as the ratio of wildtype to mutant plasmids and normalized to p850-luc and Renilla luciferase activity.

### Ligand dependent Notch activation assay

The ligand-dependent activation assay was carried out as described [26]. Briefly, urothelial cancer cell lines seeded in 6 well plates were co-transfected at 80% confluency with NOTCH1 full-length expression plasmid (or vector), the Notch-responsive reporters pJH26A or pJH28A and Renilla luciferase plasmid at a 1:1:0.1 ratio. After 24 h cells were replated in triplicates in 24 well plates coated with ligand or controls. For that purpose, the wells were first coated with 20 μg/ml goat anti-human IgG_1C_ Fc secondary antibody (ThermoFisher Scientific, Germany), washed, and then incubated for 2 h with 10 μg/ml DLL1 extracellular domain (aa 1–545) fused at its C-terminus to the Fc portion of human IgG (DLL1:Fc; AdipoGen, Switzerland) or human IgG (ThermoFisher Scientific, Germany), followed by a further wash. After a further 24 h, cells were harvested and assayed for luciferase activities by the Dual-Luciferase reporter assay (Promega). Basal activity in cells exposed to immobilized IgG was set as 1 for each cell line.

### Colony forming assay

For the colony forming assay 10^5^ cells were seeded in 6 well plates and transfected with pIRES2-N1ICD or the corresponding pIRES2 empty vector, or with pcDNA4/to-Notch1 full-length (N1-FL) or the corresponding pcDNA4/to-lacZ plasmid. After 24 h cells were seeded in triplicates into 10 cm plates and selection for stable integration of the plasmids was started the next day. Cells transfected with pIRES2 plasmids were treated with 1.0 - 1.8 mg/ml Geneticin Selective Antibiotic G418-Sulfat (Invitrogen) and cells transfected with pcDNA4/to-plasmids were treated with 100–150 μg/ml Zeocin Selection Reagent (Invitrogen) every two days for 2 weeks. After a one week recovery period with lower antibiotic concentrations, the remaining colonies were fixed with 100% methanol and stained with Giemsa solution (Sigma-Aldrich, Germany). Colonies were then counted and statistical comparisions were made by Student’s T-Test.

### RNA isolation and RT-PCR

Total RNA was isolated from sub-confluent cell lines using the RNeasy Mini Kit (Qiagen, Hilden, Germany). RNA was reverse transcribed using 200 U SuperScriptII reverse transcriptase (Invitrogen, Darmstadt, Germany), with 300 ng oligo-dT and 25 ng random hexamer primers. Real Time-PCR assays were performed with the ABI7900HT System using the QuantiTect SYBR Green PCR Kit (Qiagen) and premade or self-designed primers (Additional file 1: Tables S1–S2). *TBP* was used as a reference gene. Statistical comparisons were made by Mann–Whitney U test with SPSS 21.

### Immunocytochemistry

Cells grown to 70% confluence were fixed with 100% ice-cold methanol for 5 min, permeabilized in 0.1% Saponin-TBS solution and blocked in 1% bovine serum albumin in 0.2% Tween-TBS for 30 min each. Cells were then incubated with the primary antibodies NOTCH1 (Epitomics, EP1238Y), JAG1 (Santa Cruz Biotechnology, H114), DLL1 (Santa Cruz Biotechnology, H-265; Abcam, ab84620) and NOTCH2 (Abcam, ab8926) and subsequently with goat-anti-mouse Alexa Fluor488 antibody. Nuclei were stained with 1 μg/ml DAPI (Dako, Hamburg, Germany).

### Western blotting

Subconfluently grown cells were lysed in RIPA buffer with protease and phosphatase inhibitors. Western blot analysis after SDS-page was performed using primary antibodies against NOTCH1 (Epitomics, EP1238Y), JAG1 (Santa Cruz Biotechnology, H114), DLL1 (Santa Cruz Biotechnology, H-265; Abcam, ab84620), NOTCH2 (Abcam, ab8926) and α-tubulin (B-1-5-2, Sigma) as a control. Secondary antibodies labelled with HRP and the ECL advanced or ECL Select kits (GE Healthcare) were used for detection.

### Immunhistochemistry

Immunohistochemistry was performed by standard techniques with antigen retrieval by citrate buffer pH 9. Antibodies used were NOTCH1 (Epitomics), JAG1 (Santa Cruz) and DLL1 (Santa Cruz). Detection was achieved via a biotin-conjugated polyvalent antibody with an avidin-biotin-peroxidase reagent (Scy Tek, Logan, USA) and diamino-benzidine staining. Sections were counter-stained with hemalum. The staining results of the reference tissues for antibody specificity are provided in Additional file 1: Figure S1.

## Results

### The Notch receptor NOTCH1 and its ligand DLL1 are significantly down-regulated in urothelial carcinoma tissues

**Figure 1:**
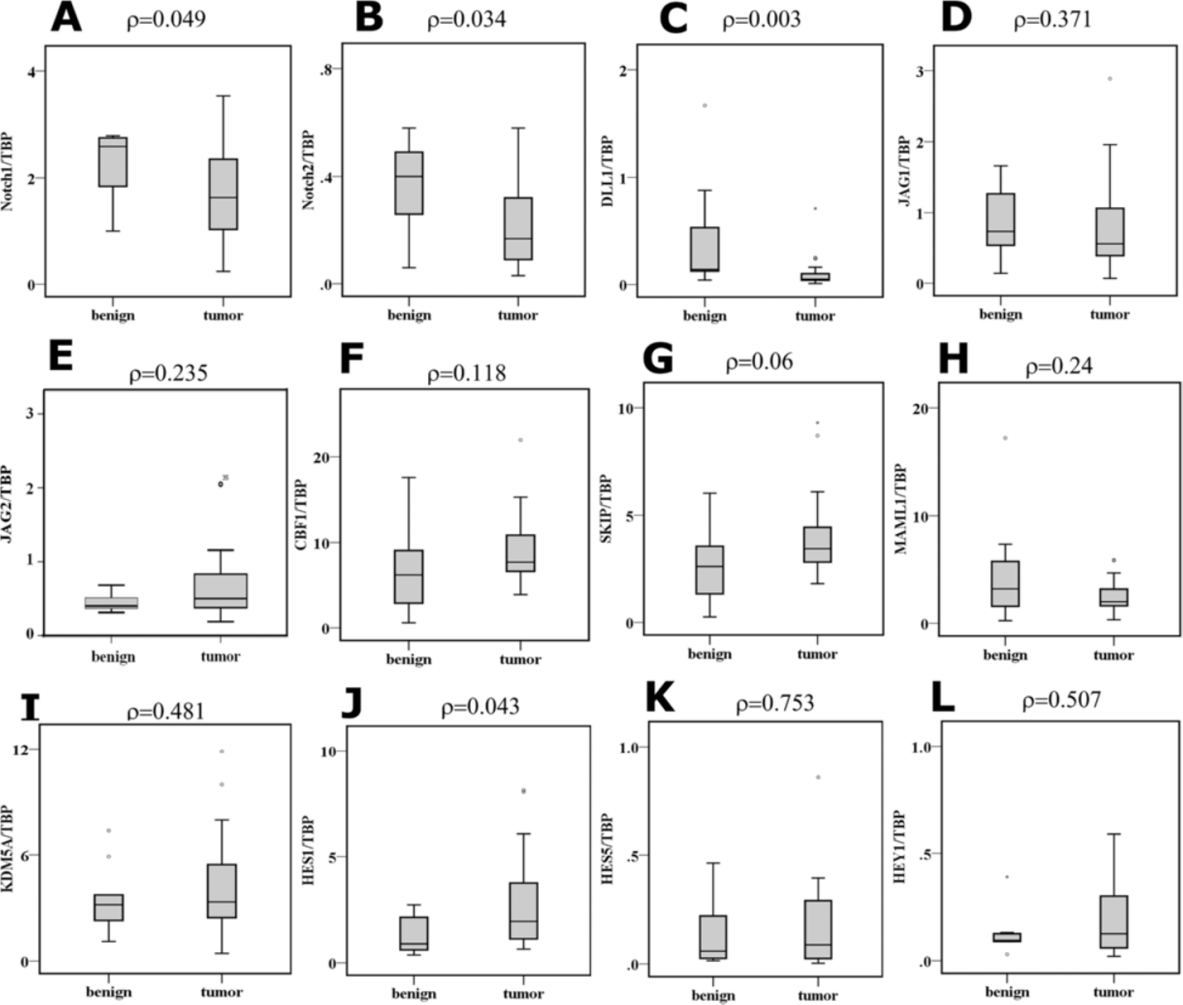
**Expression of Notch pathway components in bladder tissues.** Relative mRNA expression of Notch pathway components in 11 benign and 30 tumour bladder tissue samples was measured by qPCR for the Notch receptors **(A)** *NOTCH1*, **(B)** *NOTCH2*, for the canonical ligands **(C)** *DLL1*, **(D)** *JAG1*, **(E)** *JAG2,* nuclear factors **(F)** *CBF1,* **(G)** *SKIP*, **(H)** *MAML1*, **(I)** *KDM5A* as well as the potential Notch target genes **(J)** *HES1*, **(K)** *HES5* and **(L)** *HEY1*. Data are represented as box plots. Statistical comparisons between benign and tumour bladder tissue expression were made by the Mann–Whitney U Test using SPSS version 21.

Expression of several Notch pathway components was measured at the mRNA level by qRT-PCR in a well-characterized set of invasive urothelial carcinoma and normal bladder tissue samples that we had previously used for other studies [27]. Expression of *NOTCH1* and *NOTCH2* was significantly decreased in almost all urothelial cancers (Figure 1A-B). *NOTCH3* expression was generally unchanged in cancer tissues. *NOTCH4*, which marks endothelial cells, was strongly expressed in bladder tissues but rarely detectable in cultured cells (data not shown). Among the ligands, *DELTA1 (DLL1)* expression was strongly and significantly decreased in cancer tissues, whereas *JAGGED1 (JAG1)* and *JAGGED2 (JAG2) mRNA levels* were not significantly changed (Figure 1C-E). Expression of *DELTA3* and *DELTA4* was undetectable in normal and cancer tissues. None of the nuclear components, *KDM5A, MAML1, SKIP* and *CBF1* showed significant expression changes between normal and cancer tissues (Figure 1F-I). Expression of *HES1* and *HEY1* was rather enhanced in cancer tissues, reaching significance for *HES1*, whereas *HES5* was unchanged (Figure 1J-L).

### Expression and intracellular distribution of NOTCH1, DLL1 and JAG1 proteins is commonly changed in urothelial carcinoma

**Figure 2:**
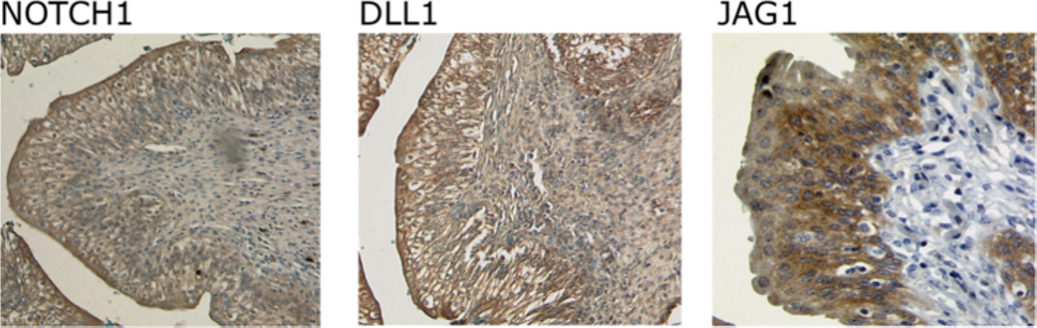
**Immunohistochemical detection of NOTCH1 and its ligands DLL1 and JAG1 in a benign bladder tissue.** In this representative benign urothelium (corresponding normal tissue from case #15) NOTCH1 and DLL1 are localized in the cytoplasm and plasma membranes of umbrella cells and the membranes predominantly of intermediate cells. Staining in the basal layer is weak, especially for NOTCH1. JAG1 is conversely detectable more strongly in the lower cell layers, mostly in the cytoplasm and weakly at the plasma membrane.

**Figure 3:**
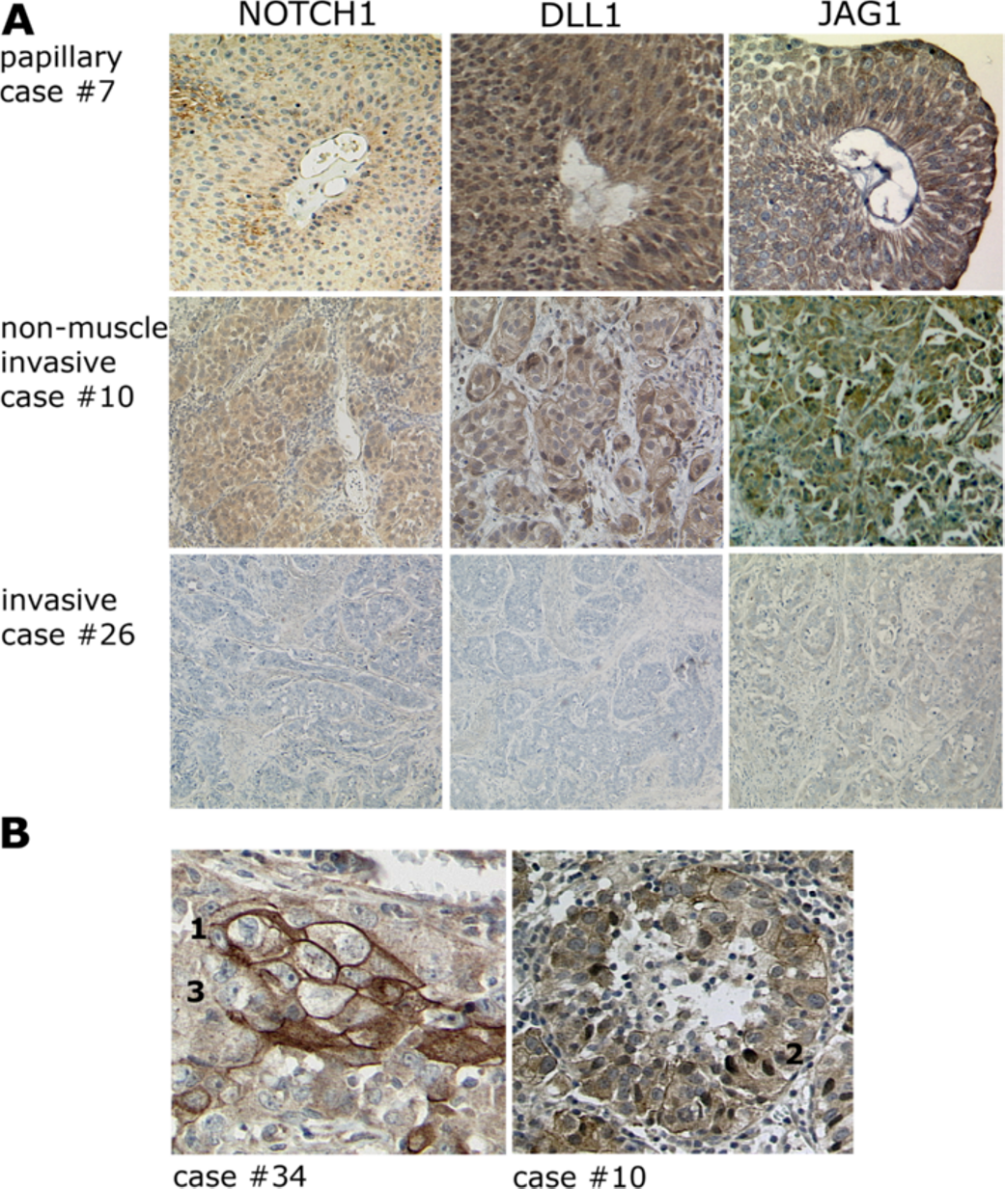
**Immunohistochemical staining of NOTCH1 and its ligands DLL1 and JAG1 in representative bladder tumour tissues. (A)** NOTCH1, DLL1 and JAG1 staining decreased with tumour invasiveness and became undetectable for NOTCH1 and JAG1 in several muscle invasive bladder tumours. **(B)** Two examples for intratumoral heterogeneity of DLL1. [1] membrane staining, [2] nuclear staining, [3] no detectable staining.

Expression of NOTCH1, DLL1 and JAG1 was further analysed by immunohistochemistry in a set of paraffin-embedded tissues covering urothelial carcinomas of all stages and grades (Table 1). In normal urothelium, NOTCH1 was predominantly localized at the membranes of intermediate cells and, more strongly, of umbrella cells. Staining of basal cells was weak. No nuclear staining was observed in UC compared to breast cancer (Additional file 1: Figure S1A). The distribution of DLL1 was very similar. JAG1 was observed in the cytoplasm and at the membranes of lower urothelial layer cells (Figure 2). Expression and intracellular distribution of the three proteins changed in urothelial carcinomas (Figure 3A). NOTCH1 and JAG1 decreased with increasing tumour stage and became undetectable in several high stage tumours. In low grade papillary tumours both proteins remained mainly associated with the plasma membrane, whereas residual protein in higher grade and stage cancers was essentially cytoplasmic. DLL1 remained expressed in low grade papillary tumours, albeit with a more cytoplasmic localization. In higher grade and stage cancers, DLL1 was diminished, often heterogeneously expressed and delocalized to the cytoplasm and focally even to nuclei (Figure 3B). An overview of the staining results is provided in Table 1.

**Table 1:** **Overview of IHC staining results in 26 urothelial cancers**

Case #	Stage	Grade	Metastasis	NOTCH1	DLL1	JAG1
1	pTa	Low	-	1	3	2
2	pTa	Low	-	0	2	2
3	pTa	Low	-	1	2	2
4	pTa	Low	-	0	2	1
6	pTa	Low	-	1	1	1
7	pTa	High	-	1	2	2
9	pTa	High	-	1	2	1
12	pTa	High	-	1	2	2
19	pTa	High	-	0	1	2
20	pTa	High	-	0	1	1
25	pTa	Low	-	1	3	2
8	CIS	High	-	1	2	2
10	pT1	High	-	1	2	2
11	pT1	High	-	1	2	2
18	pT1	High	-	0	1	1
23	pT1	High	-	0	1	1
24	pT1	High	-	0	1	1
13	pT2	High	-	0	1	1
22	pT2	High	-	0	1	1
29	pT2	High	-	0	1	1
14	pT3	High	+	0	1	1
15	pT3	High	-	1	2	2
26	pT3	High	+	0	0	0
27	pT4	High	+	0	0	1
34	pT4	High	+	0	1	1
35	pT4	High	+	1	2	1

Intensity of staining was rated by two independent observers as 3 (strong compared to normal bladder urothelium), 2 (comparable to normal), 1 (weak or very weak compared to normal), 0 (undetectable).

**Figure 4:**
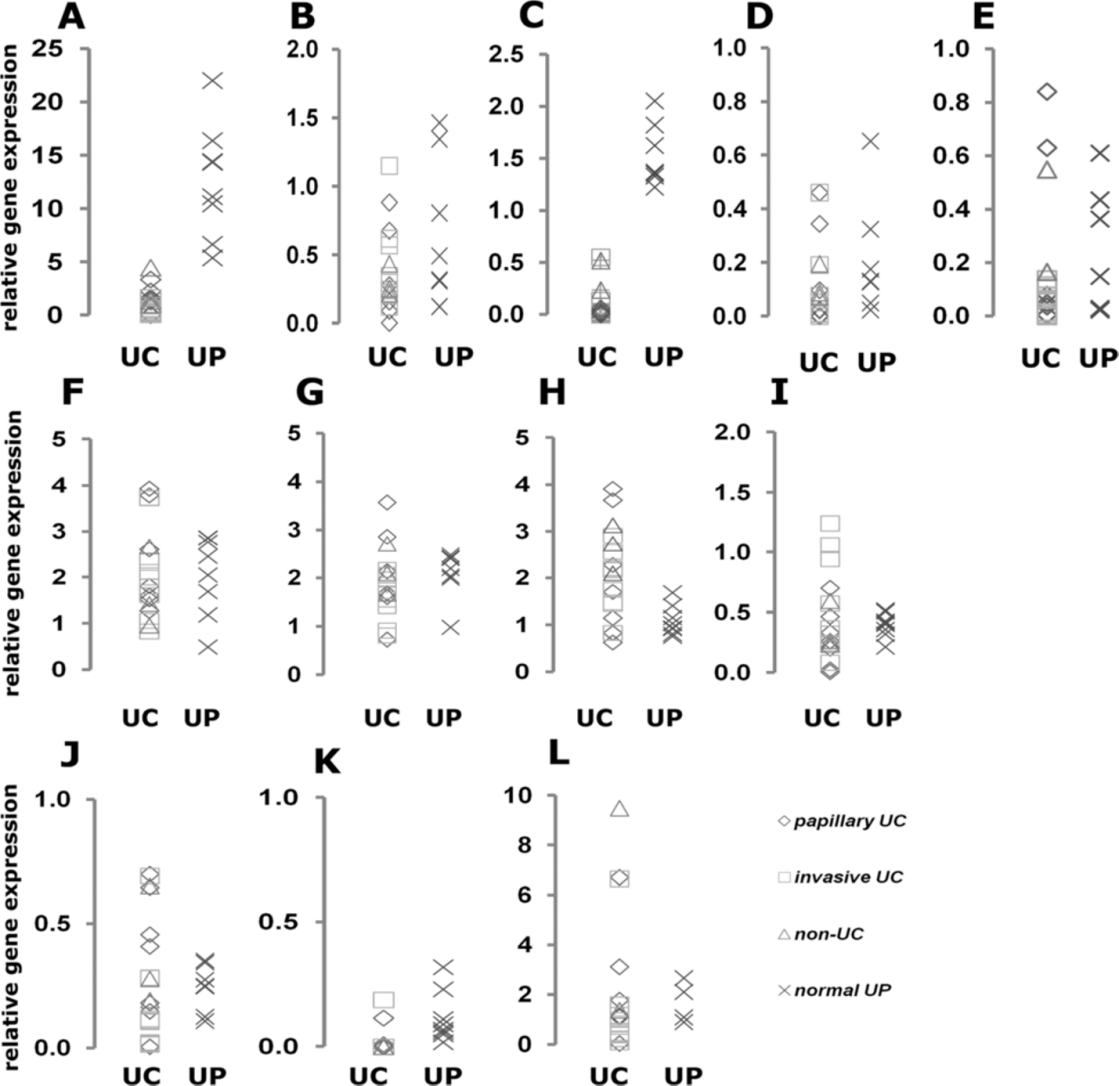
**Expression of Notch pathway components in urothelial cells.** Relative mRNA expression in 8 normal urothelial cell cultures (x) and 16 urothelial cancer cell lines, subdivided in 7 papillary UC (♦) 9 invasive UC (■) as well as 3 non-UC bladder cancer cell lines (▲), was measured by qPCR for the Notch receptors **(A)** *NOTCH1*, **(B)** *NOTCH2*, for the canonical ligands **(C)** *DLL1*, **(D)** *JAG1*, **(E)** *JAG2,* the nuclear factors **(F)** *CBF1,* **(G)** *SKIP*, **(H)** *MAML1*, **(I)** *KDM5A* as well as the potential Notch target genes **(J)** *HES1*, **(K)** *HES5* and **(L)** *HEY1*.

In UC cell lines *NOTCH1* mRNA was likewise decreased compared to normal cultured urothelial cells (UP), whereas *NOTCH2* expression was only slightly diminished. Expression of *DLL1* was strongly reduced, but that of *JAG1* and *JAG2* less so (Figure 4). No significant differences were observed for the nuclear components *CBF1* and *SKIP* whereas *KDM5A* and *MAML1* were strongly expressed in several UC cell lines. Expression of the putative target genes *HES1* and *HEY1* was increased in individual UC cell lines only.

**Figure 5:**
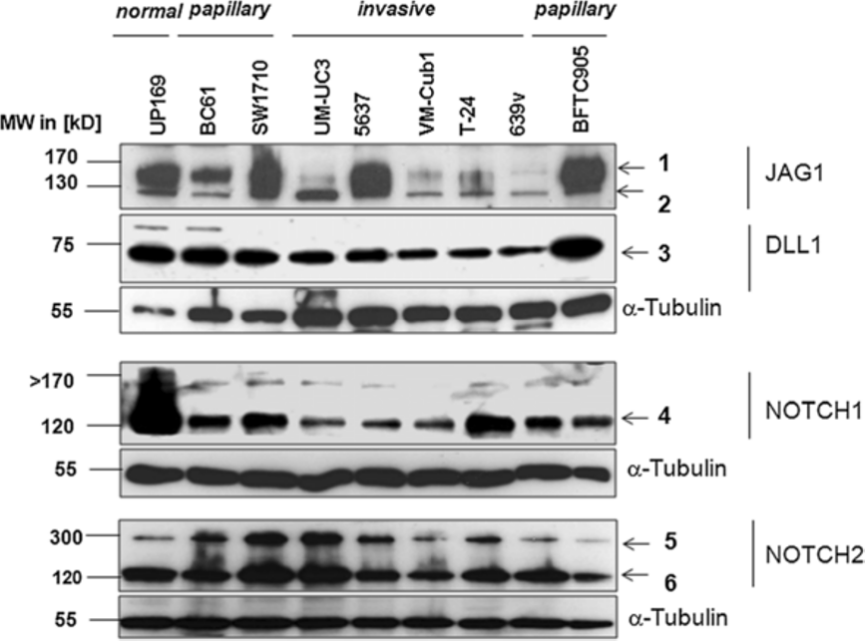
**Expression of Notch pathway component proteins in urothelial cells.** Western Blot analysis of Notch ligands and receptors in urothelial cancer cell lines compared to a primary normal urothelial cell culture (UP). Top to bottom: Analysis with a JAG1 antibody resulted in two signals at approximately [1] = 170 kD and [2] = 130 kD, likely corresponding to JAG1 full-length protein and its processed extracellular domain. Signal [2], in particular, was strongly decreased in invasive UC cell lines compared to normal urothelial and papillary UC cells. DLL1 protein was detectable at the expected size of 75 kD [3]. The signal was decreased in invasive UC cell lines compared to papillary UC and normal urothelial cells. Analysis with NOTCH1 antibody resulted in an approximately 120 kD band [4] indicating a C-terminal NOTCH1 fragment which was strongly diminished in urothelial cancer cell lines. Band [5] at ~300 kD likely corresponds to full-length NOTCH2 protein and band [6] at ~120 kD to the processed C-terminal NOTCH2 fragment. No general decrease in expression is discernible in the UC cell lines. α-Tubulin (Sigma) was used as a quality and loading control.

As in tissues, the changes in the mRNA expression of Notch pathway components in cell lines extended to the protein level (Figure 5). Normal urothelial cells (UP) expressed robust amounts of the processed cytoplasmic NOTCH1 fragment, which was depleted severely in all UC cell lines. DLL1 was likewise more strongly expressed in UP and in cell lines from papillary tumours. Contrasting with the general downregulation of NOTCH1 and DLL1, JAG1 protein remained comparable between UC and normal cells. NOTCH2 signals likely corresponding to full-length NOTCH2 and N2CTD were rather increased in cell lines with a mesenchymal phenotype (Figure 5). Immunocytochemistry revealed membranous and cytoplasmic localization of NOTCH1 and JAG1 in papillary UC lines and weak cytoplasmic staining in invasive UC cells (Additional file 1: Figure S2). As in tissues, DLL1 was often heterogeneously distributed between cells with nuclear or cytoplasmic staining. NOTCH2 was predominantly localized in nuclei in papillary urothelial cell lines, but predominantly cytoplasmic in invasive urothelial cell lines.

### Canonical Notch signalling is inactive, but remains inducible by NOTCH1 in urothelial carcinoma cell lines

**Figure 6:**
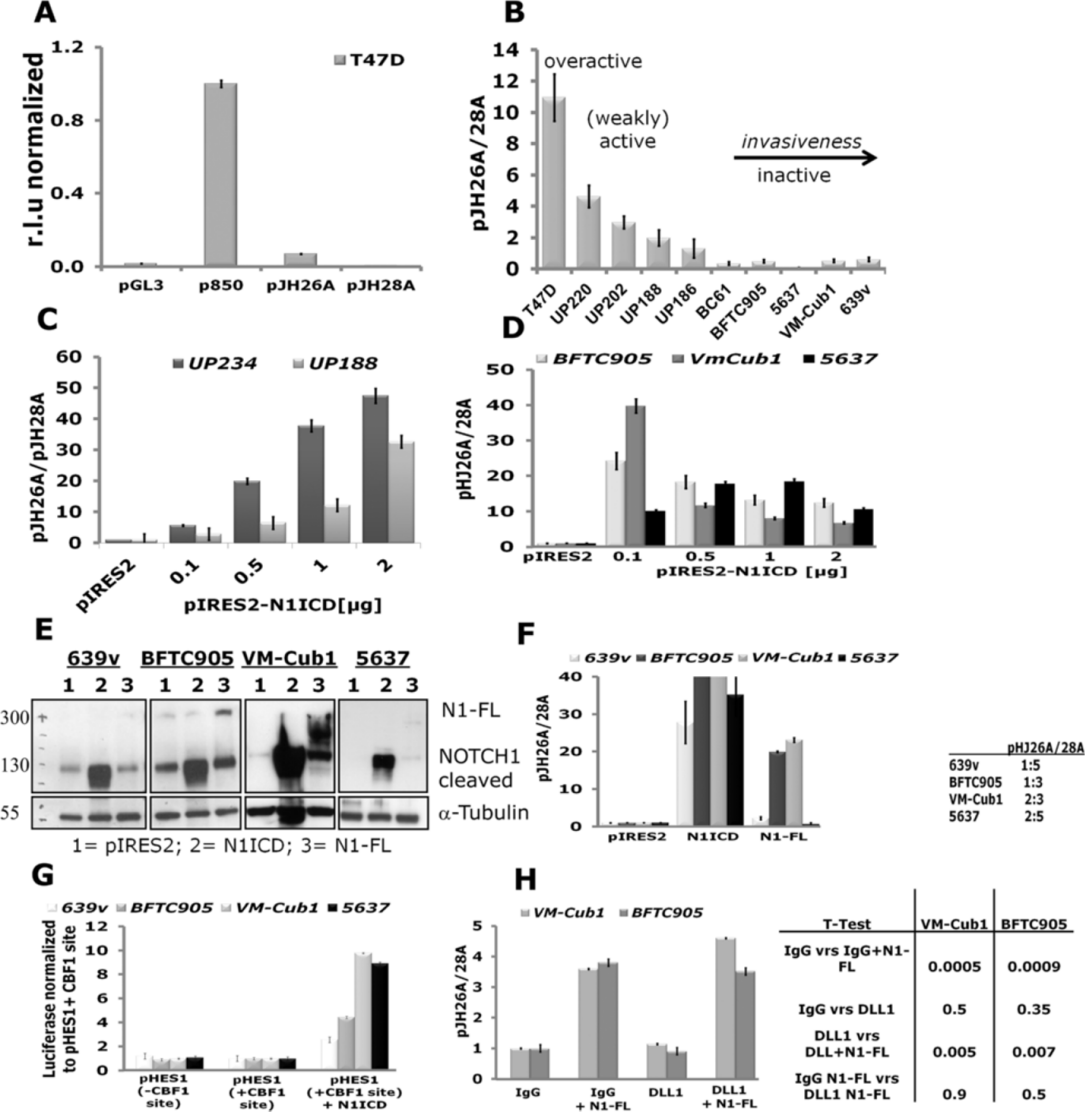
**Notch signalling pathway activity in urothelial cells.** Notch activity was determined by the ratio of the Notch-dependent wildtype plasmid pJH26A to the Notch-independent mutant plasmid pJH28A. p850-luc and pGL3 plasmids were used as controls. **(A)** The mammary carcinoma cell line T47D showed a ratio 11:1 suggesting Notch activity. **(B)** Results of reporter gene assays in primary urothelial cell cultures (UP) and urothelial cancer cell lines in comparison to T47D. **(C-D)** Induction of Notch activity after transfection of pIRES2-N1ICD in **(C)** two normal urothelial cell cultures (UP234 and UP188) or **(D)** UC cell lines BFTC905, VM-Cub1 and 5637. In contrast to the UP cultures, in UC lines Notch activity decreased upon further increasing plasmid concentration (light grey BFTC905, dark grey VM-Cub1, black 5637). **(E)** Successful overexpression of N1ICD and NOTCH1 full-length (N1-FL) plasmids after 24 hours in UC lines proven by Western Blot with a Notch antibody detecting the C-terminal fragment (Epitomics) with α-Tubulin as a loading control **(F)** inducing Notch activity in reporter assays. pJH26A to pJH28A ratios are normalized to the empty vector pIRES2. **(G)** Reporter gene assay with a HES1 promoter with intact or without CBF1 binding sites driving luciferase expression in the four UC lines. Basal luciferase activities were not significantly different between the reporters, whereas co-transfection of the N1ICD fragment significantly activated the HES1 promoter. **(H)** Assay for DLL1-dependent activation of the Notch signalling pathway in VM-Cub1 and BFTC905 cells. Notch pathway activity was measured by reporter plasmids cotransfected with or without full-length NOTCH1 (N1-FL) and exposed to immobilized extracellular DLL1 domain or IgG. Changes in the pJH26A/pJH28A reporter ratio were not significant between IgG vs. DLL1 exposure (independent of N1-FL), whereas induction by N1-FL was highly significant (T-tests p < 0.01) in each case.

To measure canonical Notch signalling activity at its final step in the nucleus, we applied a pair of reporter gene plasmids with multiple binding sites for CBF1 functional in pJH26A, but mutated in pJH28A. Their activity ratio reflects the nuclear output of the pathway. As a positive control, the mammary carcinoma cell line T47D yielded a pJH26A/28A ratio of 11:1 indicating the expected high pathway activity (Figure 6A). UC cell lines showed ratios between 1:2 and 1:5 (Figure 6B) indicating a repressed state. The ratios in normal UP varied between individual cultures from weak repression (1:2) to moderate activation (3:1). Co-transfection of a plasmid expressing active N1ICD further increased Notch nuclear signalling in normal UPs (Figure 6C) and also in the UC cell lines showing that the Notch signalling pathway can in principle be activated by overexpression of NOTCH1. However, Notch reporter activation increased with higher amounts of plasmid in UPs, but not in carcinoma cells (Figure 6D), suggesting a toxic effect or strong negative feedback factors in the latter. More moderate activation was achieved by transfecting a plasmid expressing unprocessed NOTCH1 (N1-FL). Expression of both N1ICD and N1-FL after transfection was verified by western blotting (Figure 6E-F).

To further investigate Notch signalling at the transcriptional level, we additionally applied another pair of reporters containing the natural *HES1* promoter with intact or mutant CBF1 binding sites. As shown in Figure 6G, both HES1 reporters yielded comparable luciferase activity under basal conditions in four different UC cell lines, indicating inactive nuclear Notch signalling. Co-transfection of the active N1ICD domain activated the wildtype promoter in all four cell lines.

To obtain an indication whether lack of ligand expression contributed to Notch pathway inactivity, we investigated the effect of extraneous DLL1 ligand, as this ligand was most prominently reduced at mRNA and protein levels in UC tissues and cell lines. For that purpose, we employed an experimental setup, in which the extracellular domain of the DLL1 ligand is immobilized as a Fc-fusion protein to cell culture plates through anti-Fc-antibodies [26]. Two urothelial cancer cell lines, BFTC905 and VM-Cub1, transfected with full-length NOTCH1 or vector were seeded on the coated plates and Notch pathway activity was determined via co-transfected Notch reporter plasmids pJH26A and pHJ28A. As in the previous experiments, Notch signalling was induced by transfection of full-length NOTCH1, but neither further enhancement nor increased basal activity were achieved by exposure to the immobilized extracellular DLL1 domain (Figure 6H). This finding suggests that NOTCH1 is the most limiting factor for canonical Notch activity in UC cell lines.

### Inhibition of Notch signalling by GSIs does not affect UC proliferation

**Figure 7:**
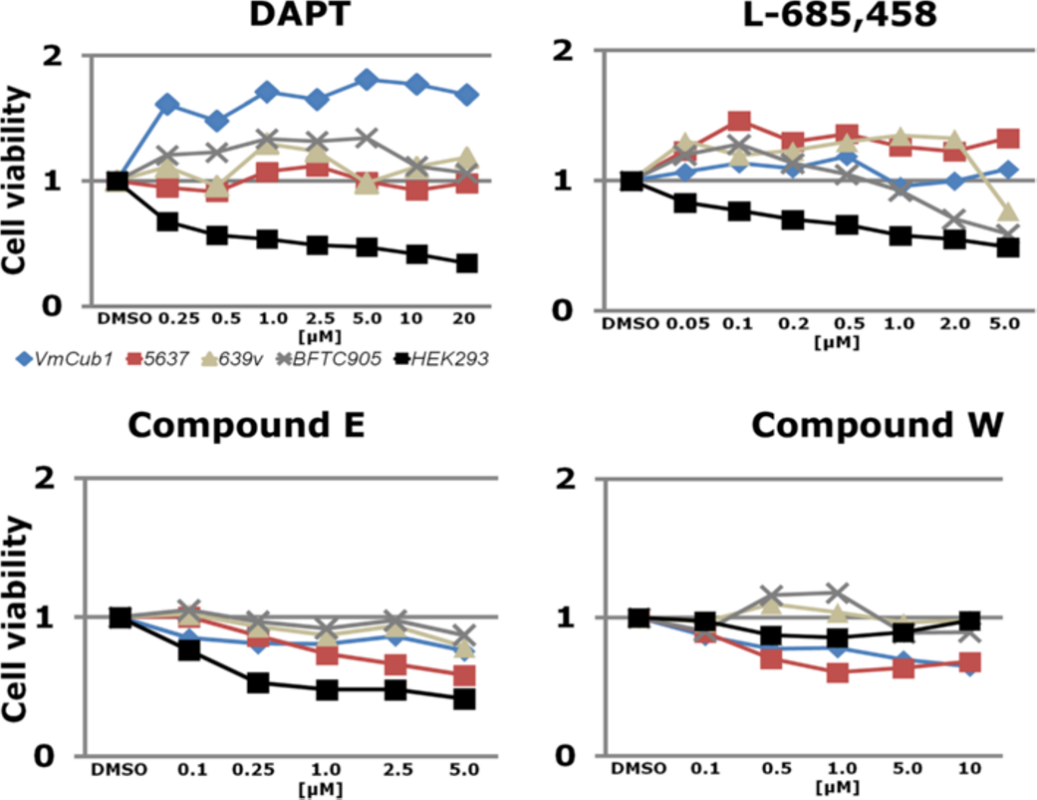
**Effects of treatment with γ-secretase inhibitors (GSI) on UC proliferation.** The indicated UC cell lines and HEK293 cells were treated with the indicated concentrations of DAPT, L-685,458, Compound E and the modulator Compound W for 48 hours after which cell viability was analysed by a MTT-Assay.

If canonical Notch signalling is indeed inactive in UC, inhibition of the essential γ-secretase cleavage should not affect cell proliferation. Therefore, we analysed the effects on cell proliferation and cell survival after pharmacological γ-secretase inhibition in urothelial cells. Three inhibitors, i.e. DAPT, L-685,458 and Compound E, inhibit the protease presenilin forming part of γ-secretase in general, whereas Compound W specifically blocks the processing of one substrate (APP), providing a specificity control. Indeed, in HEK293 cells which are sensitive to inhibition of Notch signalling, the former three inhibitors, but not Compound W, inhibited proliferation in a dose-dependent manner (Figure 7). In contrast, the specific γ-secretase inhibitors did not inhibit proliferation of the UC cell lines better than Compound W. Rather, DAPT and L-685,458 tended to increase vital cell numbers.

### Notch overexpression results in nuclear abnormalities and diminishes clonogenicity

**Figure 8:**
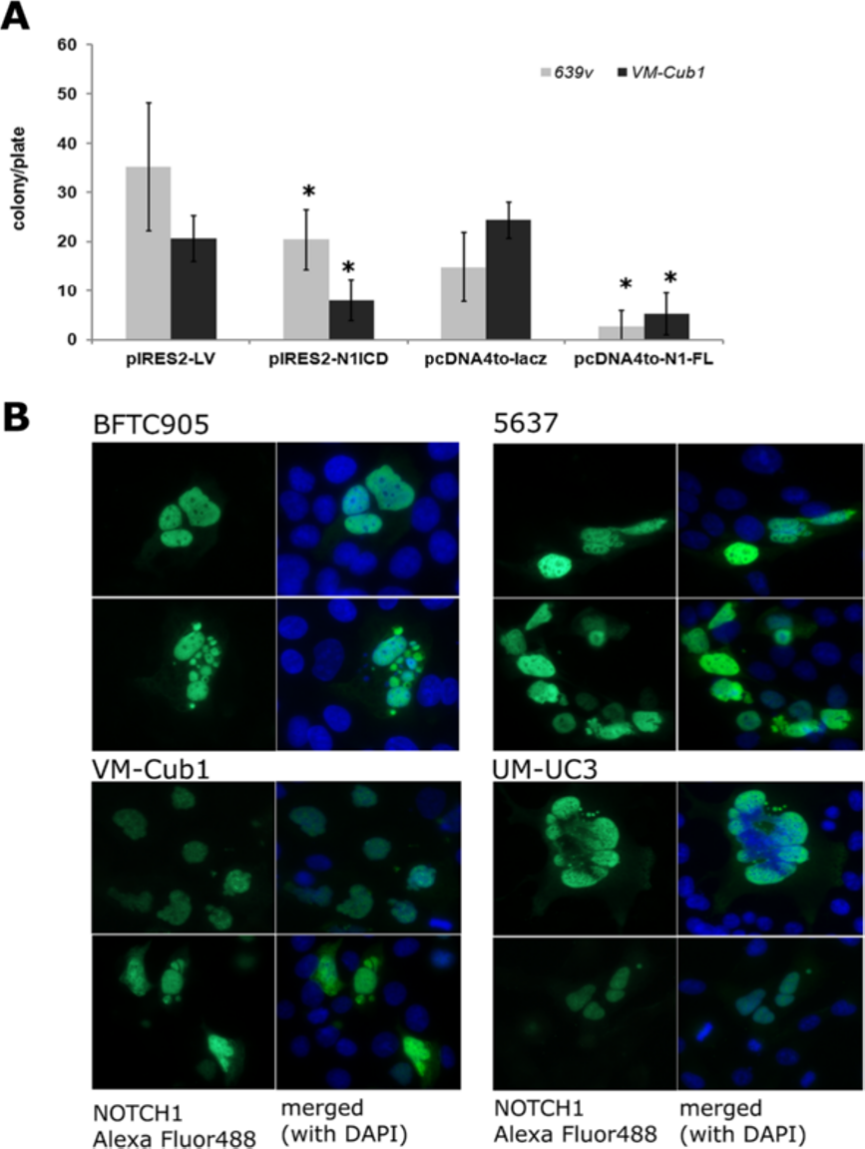
**Effects of NOTCH1 re-expression on UC cell lines. (A)** Five independent colony forming assays with triplicate plates for each experiment after transfection of plasmid constructs expressing NOTCH1 intracellular domain (N1ICD) or full-length protein (N1-FL) compared to control constructs. Clone numbers were counted after selection for three weeks with either G418 (pIRES-N1ICD/pIRES-2) or Zeozin (pcDNA4to-N1-FL/pcDNAto-lacZ). The student’s T Test was carried out for statistical analysis. Colony forming potential was significantly decreased in N1ICD and N1-FL transfected cells. **(B)** Nuclear abnormalities typically appearing after transfection of N1ICD into UC cell lines. Note nuclear enlargements, deformation, micronuclei, fragmentation, multinuclearity and mitotic catastrophe in cells expressing N1ICD (green) as compared to others. DNA was counterstained with DAPI (blue). Further analysis is shown in Additional file 1: Table S4.

Whereas pharmacological Notch signalling inhibition did not diminish proliferation in urothelial cancer cell lines, overexpression of N1-FL or N1ICD diminished clonogenicity of urothelial carcinoma cell lines (Figure 8A). In short term transfections, neither protein induced substantial apoptosis or cell cycle arrest. Accordingly, the number of transfected cells in four cell lines declined only weakly over three days by at most 45% (data not shown). However, between 35% and 64% of N1ICD-transfected cells displayed various striking nuclear abnormalities compared to controls, especially a tendency towards multinuclearity and instances of mitotic catastrophes and nuclear fragmentation (Figure 8B).

## Discussion

Taken together, our results from tissues and cell lines suggest that canonical Notch signalling towards the nucleus is inactive in urothelial carcinoma, especially in the invasive subtype. NOTCH1 and DLL1 were most consistently diminished in both cell lines and tissues. Downregulation of these factors is in accord with a previous report [19], whereas another paper reported decreased NOTCH1 expression in high-grade invasive UC tissues only, with more pervasive cytoplasmic localization [28]. The mechanisms underlying these changes remain to be determined. Sequencing data from the TCGA consortium (http://cancergenome.nih.gov/) indicate only few mutations in Notch pathway components in urothelial carcinoma (Additional file 1: Table S3). The frequency of NOTCH1 mutations is about 5% and is thus considerably lower than in head-and-neck squamous cell carcinomas [16]. A more likely cause of downregulation are copy number changes at chromosome 9q34 (where *NOTCH1* is encoded) which occur in up to 30% of invasive UC. However, neither mutations nor gene loss can fully account for the high prevalence of *NOTCH1* downregulation. As both, NOTCH1 and DLL1, are predominantly localized in more differentiated cells in the normal urothelium, their decreased expression in tumours may conceivably reflect to some extent the loss of this differentiated cell population.

In addition to downregulation, we frequently observed delocalization of NOTCH1 and DLL1 to the cytoplasm in tumour cells. Delocalization of NOTCH proteins to the cytoplasm in some cells reflects lack of activation by external ligands in trans but enables cis-inhibition by ligands expressed in the same cell compartment [29, 30]. Candidates for that function are JAG1 and especially JAG2, which appear to be retained or in the case of JAG2 even upregulated in invasive UC [31]. JAG1 was mostly cytoplasmic in tumour cells and is thus unlikely to contribute to trans-signalling. Notably, the distribution of JAG1 in normal urothelium is quite similar to that of p63 [32], a factor known to maintain basal cells in many epithelia and to induce JAG1 in many instances. The co-localization of DLL1 and NOTCH1 proteins in normal urothelium suggests that DLL1 could be the physiological ligand for NOTCH1 in this tissue. However, exposure of UC cell lines to immobilized DLL1, which is considered to yield superior activation compared to soluble ligand [26] did not enhance activation of a Notch-dependent reporter by transfected NOTCH1 protein, least activate canonical signalling by itself.

We note that NOTCH2 expression was less strongly changed than that of NOTCH1 and that active N2ICD could be detected in the nuclei of some UC cell lines. Interestingly, it appeared more strongly expressed in mesenchymal UC cell lines albeit localized to the cytoplasm. Increased expression of NOTCH2 in UC cell lines with a mesenchymal phenotype as compared to lines with an epithelial phenotype has recently been reported by others [33, 34]. The same authors observed that NOTCH2, but not NOTCH1, favors epithelial-mesenchymal transition and cell migration in UC cell lines [33, 34]. Collectively, these findings suggest divergent functions of NOTCH1 and NOTCH2 in UC, which have also been observed in other cancers [35, 36]. Nevertheless, despite the expression of N2CTD at a level comparable to that in normal urothelial cells, two different NOTCH-dependent reporters showed no evidence for canonical pathway activity in the UC cell lines. Likewise, γ-secretase inhibitors did not block cell proliferation in UC cell lines. These findings argue that canonical Notch signalling to the nucleus is inactivated in urothelial carcinoma. As a consequence, it seems plausible that the effects of NOTCH2 in mesenchymal UC cell lines, considering especially its cytoplasmic localisation, could be exerted by non-canonical signalling.

Across the cancer tissues, expression of HES1 mRNA was significantly increased compared to normal bladder, despite the significant downregulation of NOTCH1, NOTCH2 and DLL1. Although no upregulation of HES1 was observed in the UC cell lines compared to normal urothelial cells, this finding is somewhat puzzling, since HES1 is a common target gene of the canonical Notch pathway. However, target genes of the pathway vary strongly between tissues and even HES1 is not a universal target [18]. Moreover, HES1 is regulated by several pathways, including Hedgehog [37], ATF2 [38], and signalling through JNK1 [39]. In UC cell lines, a reporter plasmid driven by the HES1 promoter was similarly active, if the CBF sites were wild-type or mutant, supporting our conclusion that canonical Notch signalling is inactive. However, luciferase was induced by N1ICD cotransfection indicating that HES1 can be a target of an active Notch pathway in urothelial cells. Collectively, these findings suggest that the increased expression of HES1 in UC tissues could reflect its predominant regulation by a different pathway in the absence of canonical Notch signalling.

In normal urothelial cells and urothelial carcinoma cell lines, overexpression of full-length NOTCH1 or its active intracellular domain induced canonical reporters but was not compatible with long-term proliferation of UC cells. Inhibition of proliferation did not appear to occur by straightforward inhibition of cell cycling or by a major induction of apoptosis. Rather, NOTCH1 transfected cells often showed misshaped or abnormally large nuclei or became obviously binuclear. Intriguingly, in normal urothelium NOTCH1 is localized in the upper layers together with its likely ligand DLL1, where they might contribute to terminal differentiation. In particular, umbrella cells of the urothelium are usually polyploid [40]. In squamous epithelia, Notch signalling is most important for establishing the commitment to terminal differentiation in the suprabasal layer [41, 42]. Notch signalling is well-established as a regulator of polyploidization in Drosophila, but may also act in this fashion in certain mammalian cells [43]. Evidently, the urothelium is one tissue, where this function should be investigated in detail. The observed effects of NOTCH1 overexpression on UC cells may thus be conceptualized by assuming that it induces a failed attempt at differentiation. Evidently, the effects of pharmacological inhibition of the Notch pathway and genetic inactivation of individual components such as NOTCH1, DLL1 and NOTCH2 should be studied in culture and animal models of urothelial differentiation.

## Conclusions

Collectively, our findings indicate that canonical Notch signalling is lost in urothelial carcinoma mainly via inactivation of NOTCH1, which in normal urothelium may promote specific steps of urothelial differentiation. As it has been shown that inhibition of the γ-secretase leads to hyperproliferation in tissues, where Notch signalling promotes differentiation [9], inhibition of Notch does therefore not appear to represent a useful therapeutic approach to UC. Instead, urothelial differentiation might become disturbed as an adverse effect of long-term treatment of other tumour entities with drugs targeting canonical NOTCH signalling.

## Electronic supplementary material

Additional file 1: Figure S1: Immunohistochemical staining of reference tissues. **Figure S2.** Immunocytochemical staining of NOTCH1, NOTCH2, DLL1 and JAG1 in UC cell lines. **Table S1.** Self-designed primer assays for qPCR analysis. **Table S2.** QuantiTect primer assays from Qiagen for qPCR analysis. **Table S3.** Mutations in Notch receptors, ligands and ubiquitin ligase in urothelial cancer based on the TCGA study. **Table S4.** Statistical analysis of changes in N1ICD transfected cell lines. (DOCX 9 MB)

## References

[1] Castillo-Martin M, Domingo-Domenech J, Karni-Schmidt O, Matos T, Cordon-Cardo C (2010). Molecular pathways of urothelial development and bladder tumorigenesis. Urol Oncol.

[2] Goebell PJ, Knowles MA (2010). Bladder cancer or bladder cancers? Genetically distinct malignant conditions of the urothelium. Urol Oncol.

[3] Babjuk M, Burger M, Zigeuner R, Shariat SF, van Rhijn BW, Comperat E, Sylvester RJ, Kaasinen E, Bohle A, Palou Redorta J, Roupret M (2013). EAU guidelines on non-muscle-invasive urothelial carcinoma of the bladder: update 2013. Eur Urol.

[4] Gakis G, Efstathiou J, Lerner SP, Cookson MS, Keegan KA, Guru KA, Shipley WU, Heidenreich A, Schoenberg MP, Sagaloswky AI, Soloway MS, Stenzl A (2013). ICUD-EAU International Consultation on Bladder Cancer 2012: radical cystectomy and bladder preservation for muscle-invasive urothelial carcinoma of the bladder. Eur Urol.

[5] Pal SK, Milowsky MI, Plimack ER (2013). Optimizing systemic therapy for bladder cancer. JNCCN.

[6] Ross JS, Wang K, Al-Rohil RN, Nazeer T, Sheehan CE, Otto GA, He J, Palmer G, Yelensky R, Lipson D, Ali S, Balasubramanian S, Curran JA, Garcia L, Mahoney K, Downing SR, Hawryluk M, Miller VA, Stephens PJ (2014). Advanced urothelial carcinoma: next-generation sequencing reveals diverse genomic alterations and targets of therapy. Mod Pathol.

[7] Sternberg CN, Bellmunt J, Sonpavde G, Siefker-Radtke AO, Stadler WM, Bajorin DF, Dreicer R, George DJ, Milowsky MI, Theodorescu D, Vaughn DJ, Galsky MD, Soloway MS, Quinn DI (2013). ICUD-EAU International Consultation on bladder cancer 2012: chemotherapy for urothelial carcinoma-neoadjuvant and adjuvant settings. Eur Urol.

[8] Dikic I, Schmidt MH (2010). Notch: implications of endogenous inhibitors for therapy. BioEssays.

[9] Shao H, Huang Q, Liu ZJ (2012). Targeting notch signaling for cancer therapeutic intervention. Adv Pharmacol.

[10] Wang J, Sullenger BA, Rich JN (2012). Notch signaling in cancer stem cells. Adv Exp Med Biol.

[11] Lin C, Zheng H, Wang C, Yang L, Chen S, Li B, Zhou Y, Tan H, Li Y (2012). Mutations increased overexpression of Notch1 in T-cell acute lymphoblastic leukemia. Cancer Cell Int.

[12] Clay MR, Varma S, West RB (2013). MAST2 and NOTCH1 translocations in breast carcinoma and associated pre-invasive lesions. Human Pathol.

[13] Tonon G, Modi S, Wu L, Kubo A, Coxon AB, Komiya T, O’Neil K, Stover K, El-Naggar A, Griffin JD, Kirsch IR, Kaye FJ (2003). t(11;19)(q21;p13) translocation in mucoepidermoid carcinoma creates a novel fusion product that disrupts a Notch signaling pathway. Nature Genet.

[14] Wang NJ, Sanborn Z, Arnett KL, Bayston LJ, Liao W, Proby CM, Leigh IM, Collisson EA, Gordon PB, Jakkula L, Pennypacker S, Zou Y, Sharma M, North JP, Vemula SS, Mauro TM, Neuhaus IM, Leboit PE, Hur JS, Park K, Huh N, Kwok PY, Arron ST, Massion PP, Bale AE, Haussler D, Cleaver JE, Gray JW, Spellman PT, South AP (2011). Loss-of-function mutations in Notch receptors in cutaneous and lung squamous cell carcinoma. Proc Natl Acad Sci U S A.

[15] Agrawal N, Jiao Y, Bettegowda C, Hutfless SM, Wang Y, David S, Cheng Y, Twaddell WS, Latt NL, Shin EJ, Wang LD, Wang L, Yang W, Velculescu VE, Vogelstein B, Papadopoulos N, Kinzler KW, Meltzer SJ (2012). Comparative genomic analysis of esophageal adenocarcinoma and squamous cell carcinoma. Cancer Discov.

[16] Pickering CR, Zhang J, Yoo SY, Bengtsson L, Moorthy S, Neskey DM, Zhao M, Ortega Alves MV, Chang K, Drummond J, Cortez E, Xie TX, Zhang D, Chung W, Issa JP, Zweidler-McKay PA, Wu X, El-Naggar AK, Weinstein JN, Wang J, Muzny DM, Gibbs RA, Wheeler DA, Myers JN, Frederick MJ (2013). Integrative genomic characterization of oral squamous cell carcinoma identifies frequent somatic drivers. Cancer Discov.

[17] Egloff AM, Grandis JR (2012). Molecular pathways: context-dependent approaches to Notch targeting as cancer therapy. Clin Cancer Res.

[18] Andersson ER, Sandberg R, Lendahl U (2011). Notch signaling: simplicity in design, versatility in function. Development.

[19] Shi TP, Xu H, Wei JF, Ai X, Ma X, Wang BJ, Ju ZH, Zhang GX, Wang C, Wu ZQ, Zhang X (2008). Association of low expression of notch-1 and jagged-1 in human papillary bladder cancer and shorter survival. J Urol.

[20] Hoffmann MJ, Florl AR, Seifert HH, Schulz WA (2005). Multiple mechanisms downregulate CDKN1C in human bladder cancer. Int J Cancer.

[21] Swiatkowski S, Seifert HH, Steinhoff C, Prior A, Thievessen I, Schliess F, Schulz WA (2003). Activities of MAP-kinase pathways in normal uroepithelial cells and urothelial carcinoma cell lines. Exp Cell Res.

[22] Seifert HH, Meyer A, Cronauer MV, Hatina J, Muller M, Rieder H, Hoffmann MJ, Ackermann R, Schulz WA (2007). A new and reliable culture system for superficial low-grade urothelial carcinoma of the bladder. World J Urol.

[23] Koch A, Hatina J, Rieder H, Seifert HH, Huckenbeck W, Jankowiak F, Florl AR, Stoehr R, Schulz WA (2012). Discovery of TP53 splice variants in two novel papillary urothelial cancer cell lines. Cell Oncol.

[24] Westhoff B, Colaluca IN, D’Ario G, Donzelli M, Tosoni D, Volorio S, Pelosi G, Spaggiari L, Mazzarol G, Viale G, Pece S, Di Fiore (2009). Alterations of the Notch pathway in lung cancer. Proc Natl Acad Sci U S A.

[25] Hsieh JJ, Henkel T, Salmon P, Robey E, Peterson MG, Hayward SD (1996). Truncated mammalian Notch1 activates CBF1/RBPJk-repressed genes by a mechanism resembling that of Epstein-Barr virus EBNA2. Mol Cell Biol.

[26] Varnum-Finney B, Wu L, Yu M, Brashem-Stein C, Staats S, Flowers D, Griffin JD, Bernstein ID (2000). Immobilization of Notch ligand, Delta-1, is required for induction of notch signaling. J Cell Sci.

[27] Nikpour P, Baygi ME, Steinhoff C, Hader C, Luca AC, Mowla SJ, Schulz WA (2011). The RNA binding protein Musashi1 regulates apoptosis, gene expression and stress granule formation in urothelial carcinoma cells. J Cell Mol Med.

[28] Abdou AG, El-Wahed MM, Kandil MA, Samaka RM, Elkady N (2013). Immunohistochemical analysis of the role and relationship between Notch-1 and Oct-4 expression in urinary bladder carcinoma. APMIS.

[29] Matsuda M, Chitnis AB (2009). Interaction with Notch determines endocytosis of specific Delta ligands in zebrafish neural tissue. Development.

[30] Perez L, Milan M, Bray S, Cohen SM (2005). Ligand-binding and signaling properties of the Ax[M1] form of Notch. Mech Dev.

[31] Li W, Liu M, Feng Y, Huang YF, Xu YF, Che JP, Wang GC, Zheng JH (2013). High expression of Notch ligand Jagged2 is associated with the metastasis and recurrence in urothelial carcinoma of bladder. Int J Clin Exp Pathol.

[32] Karni-Schmidt O, Castillo-Martin M, Shen TH, Gladoun N, Domingo-Domenech J, Sanchez-Carbayo M, Li Y, Lowe S, Prives C, Cordon-Cardo C (2011). Distinct expression profiles of p63 variants during urothelial development and bladder cancer progression. Am J Pathol.

[33] Hayashi T, Gust KM, Jaeger W, Awrey S, Li N, Altamirano-Dimas M, Buttyan R, Li E, Fazli L, Black PC (2013). Specific inhibition of NOTCH-2 as a novel therapy for invasive bladder cancer. J Urol.

[34] Hayashi T, Gust K, Jaeger W, Awrey S, Li N, Altamirano-Dimas M, Buttyan R, Fazli L, Matsubara A, Black PC (2014). NOTCH2 inhibition on tumor growth and metastasis in bladder cancer. J Clin Oncol.

[35] Roy M, Pear WS, Aster JC (2007). The multifaceted role of Notch in cancer. Curr Opin Genet Dev.

[36] Ranganathan P, Weaver KL, Capobianco AJ (2011). Notch signalling in solid tumours: a little bit of everything but not all the time. Nat Rev Cancer.

[37] Ingram WJ, McCue KI, Tran TH, Hallahan AR, Wainwright BJ (2008). Sonic Hedgehog regulates Hes1 through a novel mechanism that is independent of canonical Notch pathway signalling. Oncogene.

[38] Sanalkumar R, Indulekha CL, Divya TS, Divya MS, Anto RJ, Vinod B, Vidyanand S, Jagatha B, Venugopal S, James J (2010). ATF2 maintains a subset of neural progenitors through CBF1/Notch independent Hes-1 expression and synergistically activates the expression of Hes-1 in Notch-dependent neural progenitors. J Neurochem.

[39] Curry CL, Reed LL, Nickoloff BJ, Miele L, Foreman KE (2006). Notch-independent regulation of Hes-1 expression by c-Jun N-terminal kinase signaling in human endothelial cells. Lab Invest.

[40] Biesterfeld S, Gerres K, Fischer-Wein G, Bocking A (1994). Polyploidy in non-neoplastic tissues. J Clin Pathol.

[41] Panelos J, Massi D (2009). Emerging role of Notch signaling in epidermal differentiation and skin cancer. Cancer Biol Ther.

[42] Proweller A, Tu L, Lepore JJ, Cheng L, Lu MM, Seykora J, Millar SE, Pear WS, Parmacek MS (2006). Impaired notch signaling promotes de novo squamous cell carcinoma formation. Cancer Res.

[43] Fox DT, Duronio RJ (2013). Endoreplication and polyploidy: insights into development and disease. Development.

[CR44] The pre-publication history for this paper can be accessed here: http://www.biomedcentral.com/1471-2407/14/628/prepub

