# Supplementary material for: Canonical Notch signalling is inactive in urothelial carcinoma
Source: BMC Cancer. 2014 Aug 29;14:628. doi: 10.1186/1471-2407-14-628 (PMC4242495; doi:10.1186/1471-2407-14-628)
Supplement: Supplementary file 1 — Additional file 1: Figure S1: Immunohistochemical staining of reference tissues. Figure S2. Immunocytochemical staining of NOTCH1, NOTCH2, DLL1 and JAG1 in UC cell lines. Table S1. Self-designed primer assays for qPCR analysis. Table S2. QuantiTect primer assays from Qiagen for qPCR analysis. Table S3. Mutations in Notch receptors, ligands and ubiquitin ligase in urothelial cancer based on the TCGA study. Table S4. Statistical analysis of changes in N1ICD transfected cell lines. (DOCX 9 MB) [file 12885_2014_5017_MOESM1_ESM.docx]

**Supplementary Tables**

**Table S1**: Self-designed primer assays for qPCR analysis.

| ***Gene*** | ***Primer*** | ***Sequence 5’-3’*** | ***Tm [°C]*** | ***Product [bp]*** |
| --- | --- | --- | --- | --- |
| ***CDKN1A/***  ***(P21)*** | P21_F | GGAAGACCATGTGGACCTGT | 59 | 146 |
|  | P21_R | GGCGTTTGGAGTGGTAGAAA | 57 |  |
| ***NOTCH3*** | NOTCH3_F | TGTGGACGAGTGCTCTATCG | 60 | 238 |
|  | NOTCH3_R | AATGTCCACCTCGCAATAGG | 59 |  |
| ***MAML1*** | MAML1_F1 | TACCAAGACCCGACACAA | 53 |  |
|  | MAML1_R1 | TCGAGGACAGCTGGAGTT | 56 | 114 |
| ***CBF1*** | CBF1_F1 | TGGGATAGGAAATAGTGACC | 55 | 82 |
|  | CBF1_R1 | TATATACAATGTTTTGGCTGT | 55 |  |
| ***SKIP*** | SKIP_F1 | GAGAAGAGCTGGGATCAAA | 54 | 90 |
|  | SKIP_R1 | CTCTCTTTTCGCCTGTCA | 53 |  |
| ***NOTCH1*** | NOTCH1_F | CTGGTCAGGGAAATCGTG | 56 | 95 |
|  | NOTCH1_R | AGATGTAGGAGGCCTCGA | 56 |  |
| ***TBP*** | TBP_F1 | CGAAACGCCGAATATAATCC | 55 | 62 |
|  | TBP_R1 | CGTGGCTCTCTTATCCTCA | 56 |  |
| ***KDM5A*** | KDM5A_F1  KDM5A_R1 | GCTGTGAACTTCTGTACTG  AAATTAGTTCCTCGTGTGA | 54  50 | 106 |
| ***HES1*** | HES1_F2 | GGATGCTCTGAAGAAAGATA | 53 | 132 |
|  | HES1_R2 | ACACTTGGGTCTGTGCTC | 56 |  |
| ***DLL1*** | DLL1_F | GAATGCAAGTGCAGAGTG | 58 | 148 |
|  | DLL1_R | TAGTTCAGGTCCTGGTTG | 57 |  |
| ***JAG1*** | Jagged1_F | AGGACTATGAGGGCAAGAAC | 58 | 140 |
|  | Jagged1_R | AAATATACCGCACCCCTTC | 54 |  |
| ***JAG2*** | JAGGED2_F | CCTCTGCCTTGCTACAAT | 62 | 111 |
|  | JAGGED2_R | GCACTCGTCGATGTTGAT | 64 |  |
| ***KRT14*** | CK14_F | GCGCACCATGCAGAACCTG | 61 | 140 |
|  | CK14_R | CCTCCACGCTGCCAATCATC | 61 |  |
| ***KRT5*** | CK5_F | GATGATCCAGAGGCTGAGAG | 62 | 130 |
|  | CK5_R | CTCGGCCAGCTTGTTCCTG | 61 |  |
| ***KRT20*** | CK20_F | GAACTGAGGTTCAACTAACG | 62 | 100 |
|  | CK20_R | TGGCTAACTGGCTGCTGTAA | 62 |  |
| ***UPK2*** | UPK2_F | GACAGCCACTGAGTCCAGCA | 64 | 114 |
|  | UPK2_R | AGCACCGTGATGACCACCAT | 63 |  |

**Table S2**: Primer assays from Qiagen. The annealing temperature is 55°C for all Qiagen QuantiTect qPCR assays.

| ***Gene*** | ***Assay*** | ***Primer location*** | ***product [bp]*** |
| --- | --- | --- | --- |
| ***HEY1*** | QT00035644 | Exon 4/5 | 126 |
| ***NOTCH2*** | QT00072212 | Exon 33/34 | 109 |
| ***DLL3*** | QT00021791 | Exon 2/3 | 111 |
| ***DLL4*** | QT00081004 | Exon 4-6 | 137 |

**Table S3**: Mutation status of Notch receptors (NOTCH1-4), Notch ligands (DLL1,3,4 and JAG1,2) and the ubiquitin ligase FBXW7 in urothelial cancer. This overview is based on the current TCGA study (<http://cancergenome.nih.gov/>).

| **gene** | **mutation frequency** | **affected domain(s)** |
| --- | --- | --- |
| *DLL1* | 3/104 | ECD (DSL Domain, EGF like) |
| *DLL3* | 2/104 | All ECD (EGF like) |
| *DLL4* | 0/104 | -- |
| *JAG1* | 4/104 | All ECD (DSL Domain, EGF like) |
| *JAG2* | 2/104 | All ECD (DSL Domain, EGF like) |
| *FBXW7* | 11/104 | F-Box, WD40 |
| *NOTCH1* | 5/104 | ECD (EGF like, LNR), NICD (poly-Val, poly-Ser) |
| *NOTCH2* | 6/104 | ECD (EGF like), NICD (ANK) |
| *NOTCH3* | 1/104 | NICD/poly-Val region |
| *NOTCH4* | 2/104 | ECD (EGF like) |

*Abbreviations: ECD= extracellular domain; DSL= Delta Serate Lag; EGF= Epidermal Growth Factor; LNR= Lin Notch Repeats; ANK= ankyrin domain*

**Table S4** Analysis of hN1ICD transfected cells

The amount of N1ICD transfected cells and the number of aberrant cells changed slightly but not significantly throughout 72h of analysis.

|  | **% NOTCH1 positive cells** | **% NOTCH1 positive with aberrant nuclear phenotype** |
| --- | --- | --- |
| BFTC905 | 6.5 +/- 2.1 | 50.6 +/-14.3 |
| UM-UC3 | 11.6 +/- 6.6 | 44.4 +/-17.2 |
| VM-Cub1 | 22.1 +/- 7.7 | 53.8 +/- 8.9 |
| 5637 | 28.5 +/-8.0 | 54.4 +/- 13.6 |

**Supplementary figures**

**
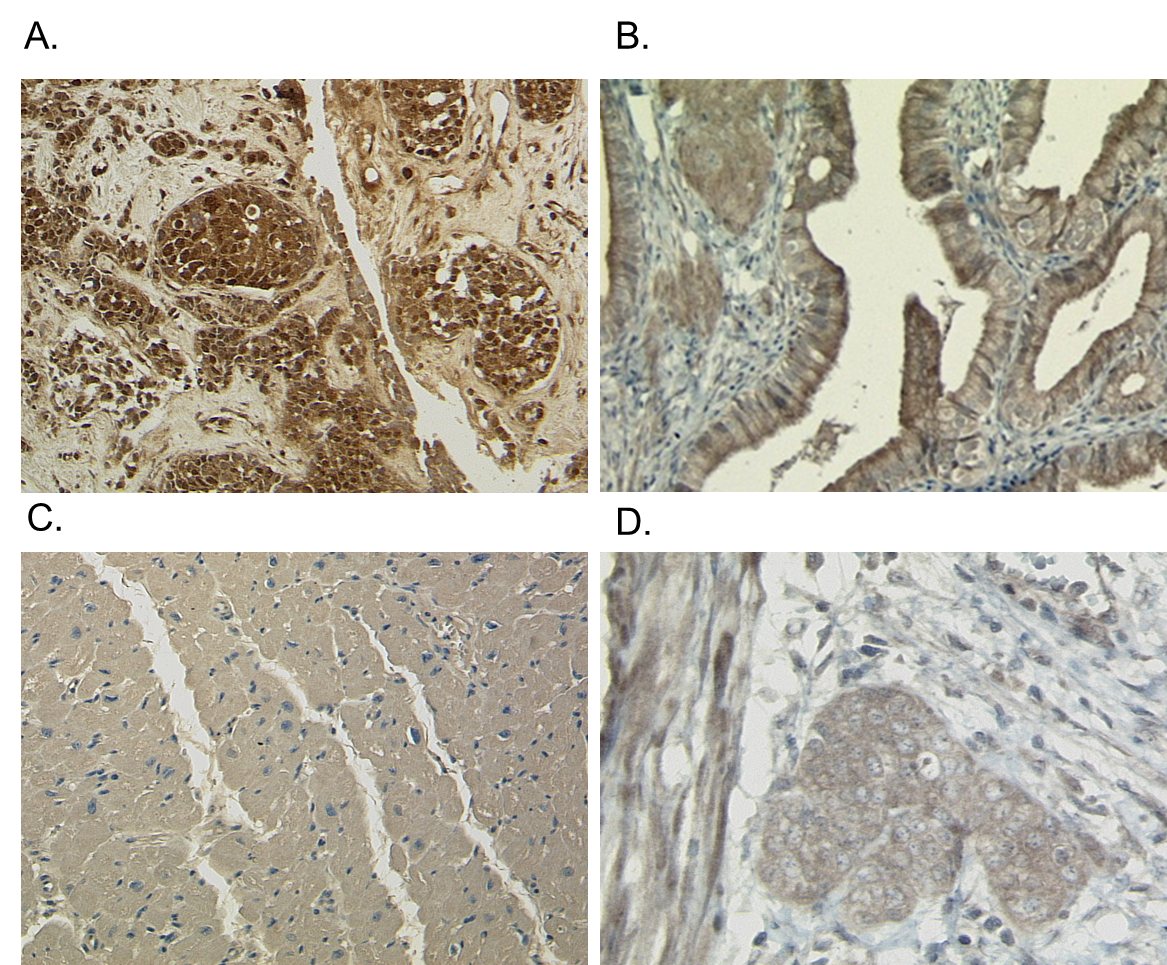
**

**Figure S1** Immunhistochemical staining of reference tissues. A. Positive nuclear and cytoplasmic NOTCH1 staining in a moderately differentiated (G2) adenocarcinoma and CIS high grade mammary carcinoma. B. JAG1 staining in the epithelium of normal gall bladder. C. Cytoplasmic staining of DLL1 in heart muscle cells. D. Smooth muscle cells in bladder as an internal positive control for DLL1 staining.

**Figure S2** Immunocytochemical staining of Notch receptors and ligands in a representative selection of urothelial cells (UP: cultured normal urothelial cells at low density, BFTC905: epithelial, from papillary UC, 5637: epithelial, from invasive UC, 639v: mesenchymal, invasive UC). NOTCH1 and JAG1 protein localization changed from a membrane-associated (papillary UC) to a diffuse cytoplasmic localization in invasive urothelial cancer cell lines. NOTCH2 protein was detectable in cytoplasm and in nuclei of papillary urothelial cancer cells. To better demonstrate the heterogeneous distribution of membrane, cytoplasmic and nuclear forms of DLL1 in the UC cell lines, pictures were not merged with DAPI staining.
